# Engineering the Thermostability of the Mono- and Diacylglycerol Lipase SMG1 for the Synthesis of Diacylglycerols

**DOI:** 10.3390/foods11244069

**Published:** 2022-12-16

**Authors:** Lilang Li, Yonghua Wang, Ruiguo Cui, Fanghua Wang, Dongming Lan

**Affiliations:** 1 School of Food Science and Engineering, South China University of Technology, Guangzhou 510640, China; 2 Guangdong Youmei Institute of Intelligent Bio-Manufacturing, Foshan 528225, China

**Keywords:** SMG1, thermostability, rational design, molecular dynamics simulation, diacylglycerol

## Abstract

Diacylglycerols (DAGs) display huge application prospectives in food industries. Therefore, new strategies to produce diacylglycerides are needed. *Malassezia globose* lipase (SMG1) could be used to synthesize DAGs. However, the poor thermostability of SMG1 seriously hampers its application. Herein, a rational design was used to generate a more thermostable SMG1. Compared with the wild type (WT), the M5D mutant (Q34P/A37P/M176V/G177A/M294R/ G28C-P206C), which contains five single-point mutations and one additional disulfide bond, displayed a 14.0 °C increase in the melting temperature (*T*_m_), 5 °C in the optimal temperature, and 1154.3-fold in the half-life (*t*_1/2_) at 55 °C. Meanwhile, the specific activity towards DAGs of the M5D variant was improved by 3.0-fold compared to the WT. Molecular dynamics (MD) simulations revealed that the M5D mutant showed an improved rigid structure. Additionally, the WT and the M5D variants were immobilized and used for the production of DAGs. Compared with the WT, the immobilized M5D-catalyzed esterification showed a 9.1% higher DAG content and a 22.9% increase in residual activity after nine consecutive cycles. This study will pave the way for the industrial application of SMG1.

## 1. Introduction

Diacylglycerol (DAG) is a structured lipid with two fatty acids ester-linked to a glycerol backbone. DAGs are of great interest due to their multiple functionalities, which enable their extensive use in the food industry. They are emulsifiers and thus could be applied in the preparation of mayonnaise, ice cream, margarine, and even sausages [1]. DAGs and monacylglycerides currently account for about 70% of the global market for food emulsifiers [2]. In addition, DAGs show health-beneficial properties due to their molecular structure and metabolic characteristics rather than triacylglycerides (TAGs) [3]. Compared to TAGs, the inclusion of DAGs in diets might reduce the accumulation of fat and prevent weight gain [4]. Remarkably, a functional cooking oil containing more than 80% DAG has been commercially produced and sold in Japan and USA to replace conventional fats and oils. This functional oil has been approved as GRAS (generally recognized as safe) by the US Food and Drug Administration [5]. Moreover, DAGs could not only be used as pro-drugs for lymphoma and Parkinson’s disease [6] but are also considered potential candidates for the treatment of cardiovascular health and systemic inflammatory diseases [1]. Various positive effects on human health make their production of further considerable interest for various applications.

DAGs could be produced chemically or enzymatically, while the enzyme-catalyzed conversion is more efficient and selective. Mono- and diacylglycerol lipases (MDLs), a unique group of lipases, display chemical selectivity against monoglycerides (MAGs) and DAGs instead of conventional TAGs [7]. The unique enzymatic properties of these enzymes have attracted considerable attention due to their application in the synthesis of DAGs [8]. However, to the best of our knowledge, only one commercial MDL (Lipase G “Amano” 50) has been available until now [9]. In our previous research, *Malassezia globose* lipase (SMG1), an MDL, was successfully employed for the synthesis of DAGs by the esterification of glycerol with oleic acid [10]. Even though a high purity of DAGs was attained, the low thermostability of SMG1 causes high economic costs in practical applications for the production of DAGs. Therefore, the protein engineering of SMG1 is needed to acquire a more thermostable catalyst of great significance for efficient DAG synthesis.

Site-directed mutagenesis based on in silico design is an attractive method to accelerate the enzyme engineering process. The Framework for Rapid Enzyme Stabilization by Computation libraries (FRESCO) is a computationally assisted method including energy calculations and molecular dynamics simulations (MD). As a fast screening/ranking strategy, it creates a relatively small variant library by in silico screening, which can then be used for experimental evaluation [11]. This approach is based on combining as many beneficial mutations as possible instead of a single or a few stabilizing mutations; although, each of them displays a small effect [12]. The FRESCO strategy has been applied to significantly enhance the thermostability of many enzymes [11,12,13]. In addition, disulfide bonds have been considered to contribute to the stability of natural proteins. Disulfide bonds stabilize proteins by helping them undergo reversible unfolding steps, as the main chain entropy of their unfolding is reduced, and they stabilize the irreversible unfolding of proteins by reducing their unfolding rate [14]. Several computer programs have been developed to predict the potential residue pairs that might form disulfide bonds based on protein structures, which makes the design of disulfide bonds more available [15,16,17,18]. The enhancement of protein thermostability has been achieved by engineering disulfide bonds [19,20,21].

In this study, our goal was to enhance the thermostability of SMG1 through a rational design by in silico prediction to acquire a more robust catalyst for the efficient synthesis of diacylglycerols. The FRESCO approach and the engineering disulfide bonds strategy were combined to improve the thermostability of SMG1. Furthermore, the synthesis of DAGs with the immobilized SMG1 M5D mutant and the WT was evaluated.

## 2. Materials and Methods

### 2.1. Plasmid, Strains, Material, and Reagent

The gene of the WT (GenBank accession no. XM_001732152.1) was synthesized and ligated into pGAPZαA to construct the expression plasmids pGAPZαA-SMG1, which was reported in our previous study [22]. *Escherichia coli* (*E. coli*) Top10 was purchased from Weidi Biotechnology (Shanghai, China), and *Pichia pastoris* (*P. pastoris*) X-33 was purchased from Invitrogen (Carlsbad, CA, USA). The protein molecular mass marker was purchased from Takara (Dalian, China). The DAGs used for enzymatic activity assays were prepared in our lab (diolein > 80%, GC determination) [23]. All other materials and reagents were of analytical grade or higher quality.

### 2.2. The Design of Mutations

Single-point mutations were designed according to the FRESCO protocol of FoldX [24], Rosetta_ddg [25], and ABACUS [26] which were based on energy calculations. The crystal structure of the WT (PDB ID: 3UUE) was used for calculations. All amino acids inside the protein were considered and mutated in silico to other amino acids, except cysteine, with the three computational tools mentioned above. For FoldX (Barcelona, Spain), the standard parameter settings were used for the calculation and repeated five times. For Rosetta_ddg, the parameter settings described by Kellogg et al. [25] in row 3 of Table 1 were used. If the predicted G_fold_ was <−5 kJ mol^−1^, the substitution would be retained for further calculation. For ABACUS, the default protocol was employed. If the ABACUS energy was <−5 units, the mutation would be selected for further screening.

To exclude incorrect predictions, potentially stabilizing mutations were further filtrated by visual inspection and MD simulation with YASARA, in accordance with the previous description [13]. The model structure of the mutant was built by FoldX and used for five independent 100 ps MD simulations with the Yamber3 force field. After energy minimization, the system was heated from 5 to 298 K over 30 ps, followed by equilibration for 20 ps, and production was performed for 50 ps. Ten snapshots of each simulation were recorded for further analysis to eliminate mutations causing steric clashes, solvent exposure of hydrophobic side chains, broken salt bridges, highly flexible backbones, and other structural problems [11].

For the design of disulfide bonds, potential disulfide bonds were predicted with Disulfide by Design 2 (DBD2) [15], SSBOND [16], BridgeD [17], and MODIP [18].

### 2.3. Site-Directed Mutagenesis

Site-directed mutagenesis was achieved by using the QuickChange^TM^ Site-Directed Mutagenesis Kit (Stratagene, La Jolla, CA, USA) according to the manufacturer’s instructions. Recombinant plasmid pGAPZαA-SMG1 was used as the template. PCR products were digested with *Dpn* I (Thermo Fisher Scientific, Waltham, MA, USA). The digested product was purified with a PCR Product Purification Kit (Shenggong, Shanghai, China), and then transformed into *E. coli* Top10. Sequence analysis was employed for the confirmation of mutations.

### 2.4. Protein Expression and Purification

Above-constructed plasmids were further transformed into *P. pastoris* strains X-33. Positive transformants were selected on yeast extract peptone dextrose (YPD) plates containing 100 μg/mL Zeocin (Life Technologies, Carlsbad, CA, USA). Strains were inoculated into 40 mL YPD medium and cultivated at 30 °C and 230 rpm. After OD_600_ reached 0.8–1.0, 40 mL broth culture was added to 360 mL YPD medium and cultivated at 30 °C and 230 rpm for 72 h. Supernatants were harvested by centrifugation at 12,000× *g* for 10 min at 4 °C.

The samples were purified with the Ni^2+^-NTA agarose (GE Healthcare, Uppsala, Sweden) column. The washing buffer was potassium phosphate buffer (20 mM, pH 6.0) containing imidazole 20 mM and 500 mM NaCl, while the elution buffer was potassium phosphate buffer (20 mM, pH 6.0) containing 300 mM imidazole and 500 mM NaCl. For the desalting of the purified sample, a desalting column (GE Healthcare, Uppsala, Sweden) was used, and the sample was finally diluted in 20 mM potassium phosphate buffer (pH 6.0). The purity of the enzyme was monitored by using 12% SDS-PAGE.

### 2.5. Determination of Melting Temperatures

The melting temperature (*T*_m_) values of the WT, single-point mutants, disulfide bond mutants, and M5D mutants were determined with the differential scanning fluorimetry (DSF) method as previously described [13]. The mixture contained 20 μL of purified protein (0.2 mg/mL) and 5 μL 50-fold diluted SYPRO Orange dye (Sigma-Aldrich; St. Louis, MO, USA). The fluorescence of the mixture was monitored by using a CFX 96 real-time PCR system (Bio-Rad, Hercules, CA, USA). The temperature was increased from 20 to 99 °C, and the heating rate was 1.75 °C/min. The wavelengths of excitation and emission were 490 and 575 nm, respectively.

### 2.6. Measurement of Disulfide Bond Numbers

For mutants containing the introduced disulfide bond, the formation of the additional disulfide bond was confirmed through a thiol titration assay to measure the free thiol content [27]. Purified protein samples (0.5 mg/mL) in 100 mM Tris-HCl-urea buffer (pH 7.0, 8 M urea) were incubated at 37 °C for 1 h. Then 1 mL sample was mixed with 50 μL β-mercaptoethanol (Shenggong, Shanghai, China) for the reduced condition or Tris-HCl-urea buffer for the non-reduced condition. To remove β-mercaptoethanol, the sample was precipitated with 2 mL 30% (*w*/*v*) trichloroacetic acid at 37 °C for 1 h, then centrifuged at 12,000× *g* and re-dissolved with 1 mL Tris-HCl-urea buffer. One milliliter of samples (reduced or non-reduced) was mixed with 15 μL 10 mM 5,5′-dithiobis (2-nitrobenzoic acid) (DTNB) (MACKLIN, Shanghai, China) and incubated at room temperature for 20 min. The absorbance was measured at 412 nm. The formation of the disulfide bond was analyzed as described by Simpson et al. [28].

### 2.7. Enzymatic Characterization of the WT and M5D Mutant

The enzyme activity was measured by using DAG oil as the substrate according to the previous description [29]. The reaction mixture contained a 4 mL substrate (25% (*v*/*v*) DAG oil was emulsified with 2% (*w*/*v*) polyvinyl alcohol solution) and a 5 mL 10 mM potassium phosphate buffer (pH 6.0), and 1 mL of the purified enzyme solution was added to start the reaction. The reaction mixture was incubated at 25 °C and 200 rpm. After reacting for 10 min, 15 mL of ethanol was added to stop the reaction, and 50 mM NaOH was used to titrate the amount of fatty acid liberated, with phenolphthalein as the indicator. One unit of activity was defined as the amount of lipase which releases 1 μmol of fatty acid from the oil per minute under the assay conditions.

To assess the half-life (*t*_1/2_), the purified enzyme was incubated at 55 °C. The incubation times of the WT were 15, 30, 45, 60, 75, 90, and 105 s, while those of the M5D mutant were 2, 4, 6, 8, 12, 24, and 36 h. Then the residual activity was examined. The optimum temperature of lipase was evaluated by determining the activity at various temperatures (20 to 55 °C). The optimum pH was evaluated by determining the activity at various pHs (4.0 to 9.0). The residual activity of lipase that was pre-incubated at different pH buffers for 24 h was examined for pH stability. The buffers used were as follows: pH 3.0–5.0, 20 mM sodium citrate buffer; pH 6.0–7.0, 20 mM sodium phosphate buffer; pH 8.0–9.0, 20 mM Tris-HCl buffer; and pH 10.0, 20 mM sodium carbonate-bicarbonate buffer.

### 2.8. MD Simulations of the WT and M5D Mutant

MD simulation was carried out by using GROMACS 5.15 with an AMBER ff99SB-ILDN-NMR force field [30]. The structures of the WT and M5D mutant were placed in a cubic box. The minimum distance between the box edges and the surface of the lipase was 1.2 nm. TIP3P water was added to fill the box, and then sufficient sodium ions were used to neutralize the charge of the system. After energy minimization, the temperature of the system was gradually increased from 0 K to 303 K over 500 ps to balance the system, followed by 100 ns MD simulations at 303 K. GROMACS tools were used for the analysis of the trajectory.

### 2.9. Immobilization of the WT and M5D Mutant

Epoxy resin ECR8285 (Purolite, South Wales, UK) was used as a carrier for the immobilization of enzymes as previously described [31]. Enzyme solution mixed with an equal volume of phosphate buffer (pH 6.0) was added to a conical flask with a lipase/support ratio of 20 mg/g resin. The flask was shaken at 30 °C and 200 rpm for 8 h. Then the immobilized enzyme was freeze-dried in a vacuum for 12 h. For the leaching study, 50 mg of the immobilized enzyme was added into 1 mL phosphate buffer (pH 6.0) and shaken at 30 °C and 200 rpm for 2 h. Then the protein content of the supernatant was measured by Bradford assay.

### 2.10. The Synthesis of DAGs with Immobilized WT and M5D Mutant

Then, immobilized enzymes were used for the synthesis of DAGs as previously described [10]. Substrates (glycerol to oleic acids of 7:1), water (4% *w*/*w*, based on total substrates), and immobilized enzyme (5% by the total weight of substrates) were mixed in a 25 mL conical flask. The mixture was incubated at 35 °C by a thermostatic air bath shaker at 200 r/min. Samples were taken periodically. The analysis of products with HPLC is as previously described. The HPLC (Waters 2695, Milford, CT, USA) was equipped with a refractive index detector (Waters 2414, Milford, USA) and a Phenomenex Luna column (4.6 mm × 250 mm, 5 μm, Phenomenex, Torrance, CA, USA).

### 2.11. Reusability Study of Immobilized WT and M5D Mutant

A reusability study of the immobilized enzyme was carried out as previously described [32]. The activity of the first batch was considered 100%, and the activity in subsequent reactions was calculated accordingly.

### 2.12. Statistical Analysis

All experiments were independently performed in triplicate, and the data were reported as the means ± standard deviations.

## 3. Results and Discussion

### 3.1. Screening and Analyzing Diverse Mechanisms of Stabilizing Mutations

Due to the thermal instability of SMG1 and the significance of enzymatic robustness for biocatalytic applications, the thermostability of SMG1 was enhanced with a computational library design. The FRESCO workflow was applied to create a library of single-point mutations. First, three computational algorithms (FoldX, Rosetta_ddg, and ABACUS) were used to predict potential mutation sites. FoldX, Rosetta_ddg, and ABACUS provided 51, 77, and 122 mutations, respectively. Then, 219 unique mutations were further screened by MD simulation and structural inspection. Finally, 80 mutations were used for experimental verification (Table S1).

In addition, disulfide bond engineering was applied to enhance the thermostability of SMG1. Potential residue pairs that might form disulfide bonds were predicted by DBD2, BridgeD, SSbond, and Modip. The following criteria were used for the design of disulfide bonds: (i) potential residue pairs located in the loop as possible; (ii) the mutation sites near the protein surface. Twenty-six disulfide bond mutants were designed for attempting to improve the thermostability of SMG1 (Table S1).

**Figure 1:**
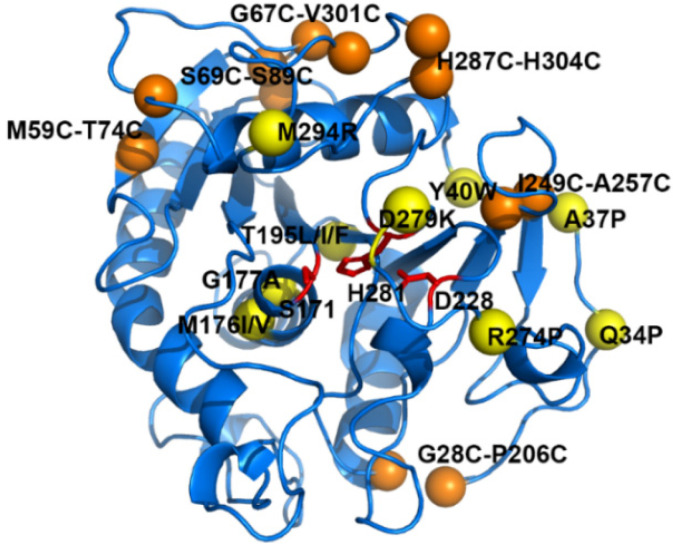
Location of stabilizing mutations in SMG1 (PDB ID: 3UUE). Yellow spheres represent effective single-point mutations and orange spheres represent effective disulfide bonds. The catalytic triad (S171, D228, and H281) is shown as red sticks.

The *T*_m_ value is an essential parameter for evaluating the thermodynamic stability of proteins. Therefore, DSF as a rapid and sensitive approach to determining *T*_m_ values of proteins was used. Through the DSF method, 12 effective single-point mutations and 6 beneficial disulfide bonds were obtained (Δ*T*_m_ ≥ 1.0 °C) (Figure 1 and Figure S1, Table 1 and Table 2). The thiol titration assay indicated that those disulfide bonds were successfully formed (Figure S2).

**Figure 2:**
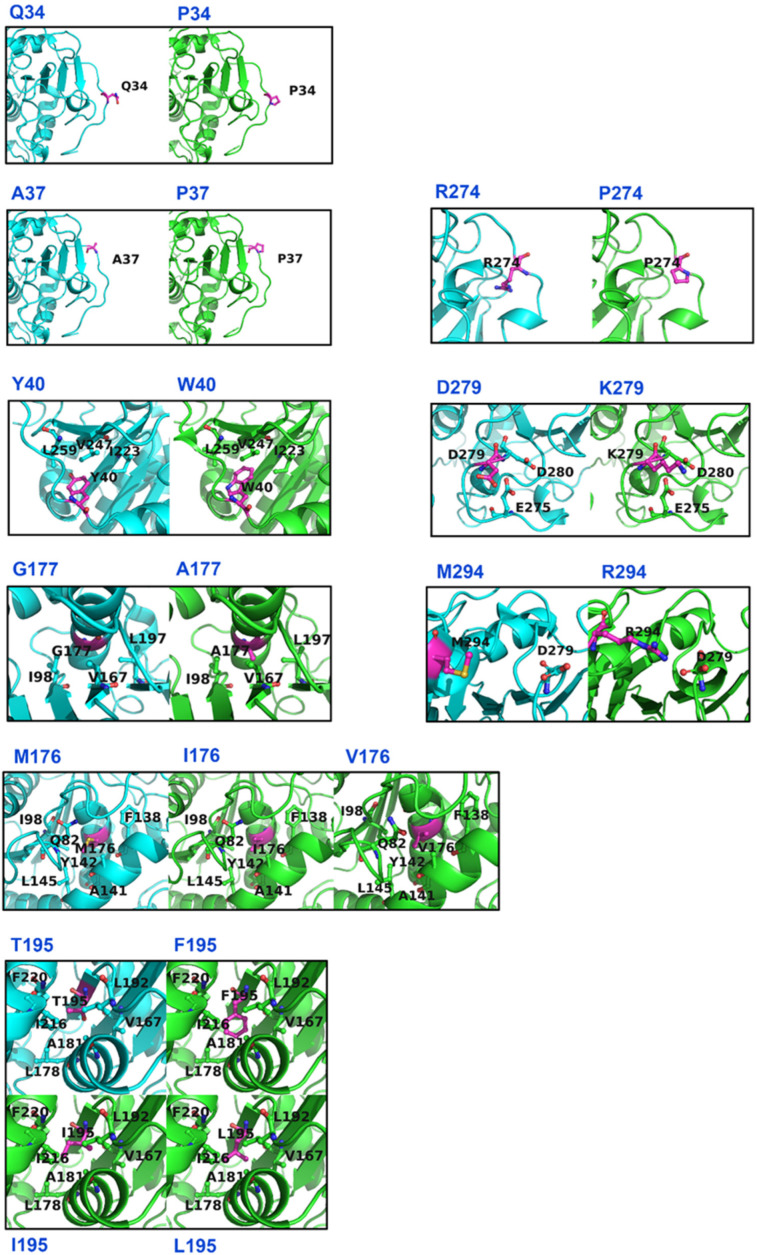
Structural effects of the stabilizing single-point mutations. The WT and mutants are shown in cyan and green cartoon, respectively. Key residues proximal to the stabilizing mutations (magenta) are shown in sticks.

Even though 12 effective single-point mutations were predicted by FRESCO, the mechanisms underlying stabilization were distinct [13]. Based on the predicted structures and snapshots during MD simulations, the main stabilization mechanisms for the effective single-point mutations were proposed as follows: reduction in the conformational entropy of local unfolded protein (Q34P, A37P, G177A, and R274P), improvement of hydrophobic packing (Y40W, M176I, M176V, G177A, T195L, T195I, and T195F), and optimal charge-charge interactions (D279K and M294R) (Table 1 and Figure 2).

Proline with a distinctive cyclic structure side chain shows a high occurrence frequency in thermophilic proteins. Its side chain can lock the backbone, contributing to the conformational rigidity of turns and loops [32]. A lower degree of proline could result in entropic stabilization in the unfolded state of the protein [33]. In our present studies, mutations Q34P, A37P, and R274P were located in loops, which might enhance the rigidity of the enzyme and reduce conformational entropy.

Hydrophobic interactions contribute about sixty percent of the thermal stability, which are critical for determining protein stability [34]. Y40W might promote hydrophobic interactions between the *N*-terminus loop and β-sheet cluster and fill the buried cavity. Other hydrophobic substitutions were located at the center of the hydrophobic core of the enzyme. M176 was replaced by more hydrophobic isoleucine/valine, facilitating the inter-helix hydrophobic interaction. For the G177A mutation, the hydrophobic substitution might cause an enhancement of hydrophobic interactions and a reduction in the unfolding entropy. Furthermore, the strongly hydrophilic hydroxyl group of T195 might lead to the destabilization of the hydrophobic packing, thus T195 substituted with hydrophobic leucine/isoleucine/phenylalanine could also strengthen the hydrophobic packing inside the enzyme.

For charged substitutions, D279K appeared to stabilize the protein by removing the negatively charged side chain, which was surrounded by the other two negatively charged amino acid residues (E275 and D280). The replacement of aspartic acid led to the reduction in repulsive electrostatic interactions in the folded state of the enzyme. Based on the predicted structures and MD simulations, M294R that improved the thermal stability of SMG1 resulted from the NH atoms of the introduced arginine, and the anti-orbitals of the carboxyl oxygen of D279 formed a new salt-bridge.

Meanwhile, six stabilizing disulfide bonds were screened in this study. The disulfide bond is a covalent link that contributes crucial stability to the protein, and each crosslink provides a range of 2.3–5.2 kcal/mol to the thermodynamic stability of the protein [35]. The enhanced stability attributes to the covalent interactions, which lead to the loss of conformational entropy of the unfolded state and reduced the unfolding rate of the protein [14]. The best disulfide bond mutant G28C-P206C (Δ*T*_m_ = 9.0 °C) might reinforce the contact between an α-helix and the loop of the *N*-terminus (Figure 1). In addition, three effective single-point mutations (Q34P, A37P, and Y40W) were also located on the *N*-terminus (Figure 1). Structurally, the *N*-terminus of SMG1 was a long loop. These results suggested that the *N*-terminus was crucial to improving the thermostability of SMG1.

### 3.2. The Combination of Stabilizing Mutations

**Figure 3:**
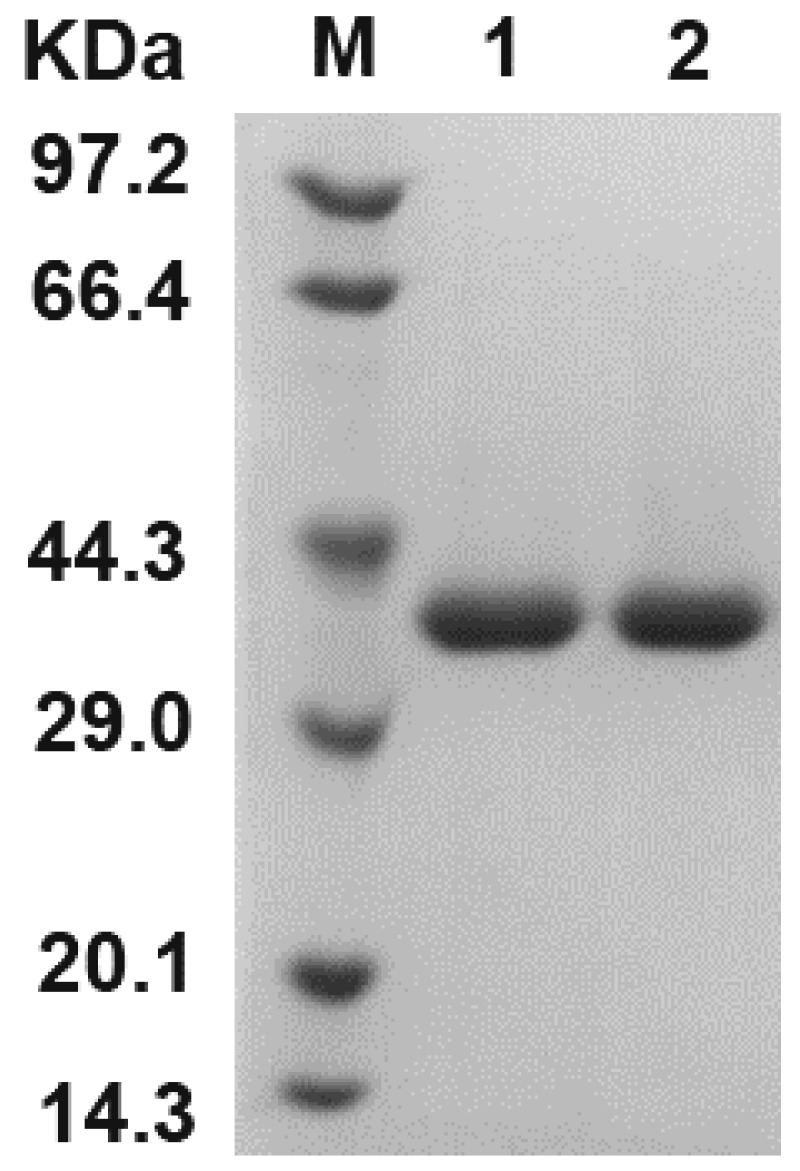
SDS-PAGE analysis of the purified WT and M5D mutant. Lane M is the molecular mass marker. Lanes 1 and 2 are the WT and M5D mutants, respectively.

To further improve the thermal stability of SMG1, the beneficial mutations were stepwise combined based on the sites, the type of secondary structure, and assumed interactions with nearby residues. We finally obtained the M5D mutant (containing five single-point mutations Q34P, A37P, M176V, G177A, and M294R, and one additional disulfide bond G28C-P206C). The purified M5D mutant (Figure 3) exhibited a 14.0 °C increase in the *T*_m_, compared to the WT (Table 3). This indicated that the thermodynamic stability of SMG1 was effectively enhanced.

Meanwhile, the stability of the WT and M5D mutant was evaluated by measuring their *t*_1/2_ values and optimum temperatures. As shown in Table 3, the WT rapidly became inactivated at 55 °C, and its *t*_1/2_ value was 1.2 min. However, the *t*_1/2_ value of M5D mutant was 1386.3 min, which increased by 1154.3 folds compared to the WT. Furthermore, the optimum temperatures of M5D mutant shifted from 25 to 30 °C (Table 3). These results indicate that the stability of the M5D mutant was improved.

Meanwhile, the specific activity of the WT and M5D mutants was evaluated. As shown in Table 3, The specific activity of the WT was 87.1 U/mg, while that of M5D mutant was 347.8 U/mg, which increased by 3.0-fold. During the process of protein engineering, the increased thermostability of the enzyme is probably accompanied by reduced activity, which is defined as the activity-stability trade-off [36]. However, both the thermostability and specific activity of the M5D mutant were increased in this study. This indicates that the great thermostability and high activity were not always contradictory.

**Figure 4:**
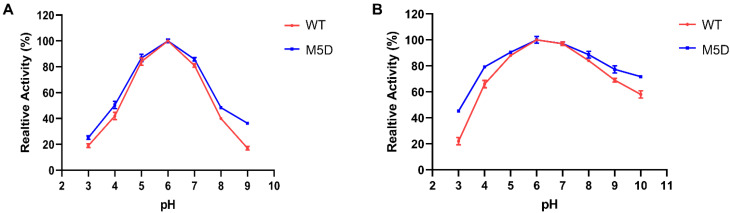
Optimal pH (**A**) and pH stability (**B**) of the WT and M5D mutants. Error bars represent standard deviations between replicates.

In addition, the effect of these mutations on SMG1 activity at different pHs was examined. As shown in Figure 4A, the WT and M5D mutants showed the same optimal pH (pH 6.0). This indicates that the M5D mutant retained the optimum pH of the WT. In addition, the M5D mutant showed about a 20% increased relative activity at pH 3 and pH 10 for the assay of pH stability (Figure 4B). This suggests the extreme pH stability of the M5D mutant.

### 3.3. MD Simulations

**Figure 5:**
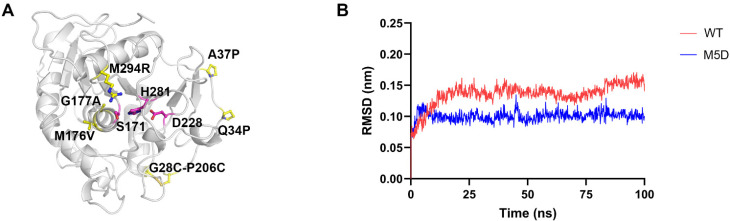
The structure of M5D mutant and MD simulation analysis of the WT and M5D mutants. (**A**) Locations of the mutations of M5D mutant. The structure of mutant M5D was built via mutagenesis program in YASARA software and the X-ray structure of the WT (PDB ID: 3UUE) was used as the template. The catalytic triad of S171, D228, and H281 is shown as magenta sticks, and mutations are shown as yellow sticks. (**B**) Cα RMSD analysis of the WT and M5D mutants at 303 K.

MD simulations were carried out to investigate the molecular basis of the M5D mutant. Based on the structure of the WT (PDB ID: 3UUE), the structure of the M5D mutant was built via the mutagenesis program in YASARA software (Figure 5A). For stability analysis, the Cα root mean square deviation (RMSD) during the MD simulation was calculated. As shown in Figure 5B, the WT exhibited a maximum RMSD of 0.15 nm, while that of the M5D mutant was below 0.10 nm. The M5D mutant displayed reduced Cα RMSD compared with the WT. The results indicate that the global structural rigidity of the M5D mutant was enhanced.

### 3.4. The Synthesis of DAGs with the Immobilized M5D Mutant

**Figure 6:**
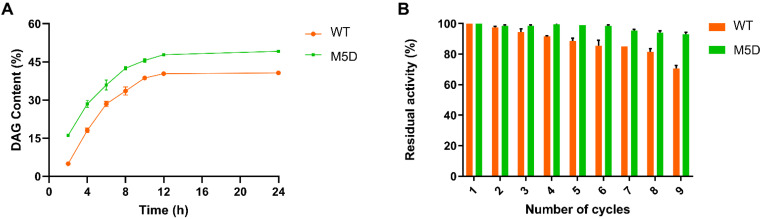
Comparison of the DAG synthesis via esterification catalyzed by the immobilized WT and M5D. (**A**) Time course of total DAG content by the immobilized WT and M5D mutants. (**B**) Operational stability of the immobilized WT and M5D mutants. Error bars represent standard deviations between replicates.

To demonstrate the practical application of the M5D mutant with better enzymatic properties, the biocatalytic synthesis of DAGs was performed. The WT and M5D mutants were immobilized onto epoxy resins (ECR8285). For the leaching study of the immobilized WT and M5D mutant, protein contents of the supernatant could not be defeminated, which indicated that the WT and M5D mutants could be tightly immobilized onto the resins. The stable immobilization might be caused by the covalent reaction between enzymes and epoxy resins [37]. Subsequently, the immobilized WT and M5D mutant were used for the DAG synthesis. As shown in Figure 6A and Figure S3, the FA conversion and the DAG content of the reaction catalyzed by the immobilized WT were 71.40% and 40.23% at 24 h, respectively, while those of the reaction catalyzed by the immobilized M5D mutant were 83.31% and 49.30% at 24 h, respectively. Compared to using the immobilized WT, the FA conversion and DAG content yielded by using the immobilized M5D mutant were increased by 11.91% and 9.07%, respectively. This might be a result of the higher stability of the M5D mutant that remained more activity during the reaction process. Zhang et al. [38] applied commercial Novozyme 435 (immobilized enzyme) to catalyze the esterification of glycerol and oleic acid, and the DAG content was 35.56% (Table S2). Li et al. [39] used Lipase G “Amano” 50 (commercial MDL lipase PCL, free enzyme) to catalyze the esterification of glycerol and soybean oil-free fatty acid, and the DAG content was 51.16%. Liu et al. [8] used immobilized MDL lipase PCL to catalyze the esterification of glycerol and α-linolenic acid, and the DAG content was 54.59%. The DAG content yielded by using immobilized mutant M5D was higher than that by using Novozymes 435 and was similar to that by using Lipase G “Amano” 50 and immobilized MDL lipase PCL in a similar reaction. It is difficult to separate TAGs from the product of esterification [40]. TAG content was 12.57% for the esterification catalyzed by Novozyme 435 [38]. However, the TAGs could not be detected in the esterification catalyzed by Lipase G “Amano” 50 and immobilized MDL lipase PCL, and our result was coincident with theirs. The above results indicate the immobilized M5D mutant might provide an alternative candidate of MDLs for the DAG synthesis.

The reusability of immobilized enzymes is critical for the cost of industrial production [41]. The reusability of the immobilized WT and M5D mutants was evaluated for nine consecutive cycles of DAG synthesis. As shown in Figure 6B, after nine cycles, the residual activity of the M5D mutant was 93.2%, while that of the WT was 70.3%, which indicated the mutant exhibited excellent reusability. This demonstrates that enhancing the thermostability of the M5D mutant could make the enzyme maintain high activity in consecutive batches. The increased reusability allowed the enzyme to maintain its activity over a more extended operational period and could bring economic benefits to the industrial DAG synthesis process. The increase in the DAG yields produced by the M5D mutant and the reusability of the M5D mutant might render the enzyme more unsuitable for the industrial synthesis of DAGs.

## 4. Conclusions

In this study, a rational design was employed to successfully improve the thermostability and activity of SMG1. Compared with the WT, the *T*_m_, optimal temperature, and half-life at 55 °C of the M5D mutant were increased by 14.0 °C, 5 °C, and 1154-fold, respectively. In addition, the M5D mutant exhibited a 3.0-fold increase in specific activity on olive oil. For the esterification of oleic acid and glycerol by using the immobilized M5D mutant, a 9.1% higher DAG content and a 22.9% increase in productivity after nine consecutive batches were gained. This suggested that the M5D mutant with enhanced enzymatic characteristics might be a suitable enzyme for the preparation of DAGs in the industrial process.

## Figures and Tables

**Table 1:** Predicted structural basis and stabilizing effect of the beneficial mutations.

Mutation	Origin	Predicted Improvement	Δ*T*_m_ (°C) ^a^
Q34P	Rosetta_ddg, ABACUS	Reduction in the conformational entropy	+2.5
A37P	FoldX, Rosetta_ddg	Reduction in the conformational entropy	+1.0
Y40W	Rosetta_ddg	Hydrophobic interactions	+1.0
M176I	ABACUS	Buried hydrophobic interactions	+1.0
M176V	ABACUS	Buried hydrophobic interactions	+1.0
G177A	Rosetta_ddg	Buried hydrophobic interactions + unfolding entropy	+2.0
T195L	ABACUS	Buried hydrophobic interactions	+2.0
T195I	ABACUS	Buried hydrophobic interactions	+1.5
T195F	ABACUS	Buried hydrophobic interactions + Filling the buried cavity	+2.0
R274P	ABACUS	Unfolding entropy	+1.0
D279K	Rosetta_ddg	Optimized surface charge + Decreased repulsive electrostatic interactions	+1.5
M294R	ABACUS	New salt-bridge interaction with D279	+1.0

^a^ ∆*T*_m_ is the difference in the melting temperature versus that of the WT.

**Table 2:** The melting temperatures of beneficial disulfide bond mutants.

Mutant	∆*T*_m_ (°C) ^a^
G28C-P206C	+9.0
M59C-T74C	+1.5
G67C-V301C	+1.5
S69C-S89C	+1.0
I249C-A257C	+1.5
H287C-H304C	+1.0

^a^ ∆*T*_m_ is the difference in the melting temperature versus that of the WT.

**Table 3:** Thermostability and specific activity of the WT and M5D mutants.

Enzyme	*T*_m_ (°C)	*T*_opt_ (°C)	*t*_1/2_ (min) ^a^	Specific Activity (U/mg) ^b^
WT	50.0	25	1.2 ± 0.2	87.1 ± 1.1
M5D	64.0	30	1386.3 ± 53.3	347.8 ± 4.2

^a^ The purified enzymes were incubated at 55 °C. ^b^ The specific activity was measured at the optimum temperature.

## Data Availability

The datasets used and/or analyzed during the current study are available from the corresponding author on reasonable request.

## Supplementary Materials

The following supporting information can be downloaded at: https://www.mdpi.com/article/10.3390/foods11244069/s1, Table S1. The list of single-point mutants and disulfide bond mutants; Table S2. Comparison of the DAG synthesis by using enzymes in this study and other reported enzymes via esterification. Figure S1. SDS-PAGE analysis of purified beneficial mutants (A) Lane M is the molecular mass marker. Lanes 1 and 12 are Q34P, A37P, Y40W, M176I, M176V, G177A, T195L, T195I, T195F, R274P, D279K, and M294R, respectively. (B) Lane M is the molecular mass marker. Lanes 1 and 6 are G28C-P206C, M59C-T74C, G67C-V301C, S69C-S89C, I249C-A257C, and H287C-H304C, respectively; Figure S2: Disulfide bond number of the WT and beneficial mutant enzymes; Figure S3. Compositions of the DAG synthesis reaction catalyzed by immobilized WT and M5D mutants.
