# Supplementary material for: Engineering the Thermostability of the Mono- and Diacylglycerol Lipase SMG1 for the Synthesis of Diacylglycerols
Source: Foods. 2022 Dec 16;11(24):4069. doi: 10.3390/foods11244069 (PMC9778158; doi:10.3390/foods11244069)
Supplement: Supplementary file 1 [file foods-11-04069-s001.zip › foods-2003936-supplementary.pdf]

foods MDPI

Supplementary materials

Table S1. The list of single-point mutants and disulfide bond mutants.

Single-point mutant			Disulfide bond mutant
G26D	W116K	Y193D	G28C-P206C
R27P	W116R	T195V	S31C-G262C
Q34P	W116Q	T195I	P35C-K258C
A37P	W116S	T195L	P35C-Y260C
N38V	Q117W [a]	T195F	N38C-A257C
N38L	N121D	G204V [a]	T42C-F286C
Y40W	I125R	G204F [a]	S46C-T288C
T42P	I125K	G204I	L47C-G67C
S46M	M135D	G204L	G50C-V301C
E61P	M135P [a]	S222Y [a]	M59C-T74C
N62P [a]	N143F	S222V	E61C-L64C
S69F	F150I	S222I	G67C-V301C
S69L [a]	Y156W	S222F	S69C-S89C
S69W	K160Y	S222L [a]	T74C-N85C
M75T	K160L	W229S	G76C-R81C
A80T	E162V	W229A	W116C-H136C
Q82P	M176V	R236P	Y127C-L186C
Q82L	M176I	D245K	Y128C-T132C
R83P	G177A	D245R [a]	K133C-N205C
H88E	C180V	A273P	A209C-Q241C
H88R	C180L	R274P	D213C-S244C
S91L	C180I	F278W	I249C-A257C
A95V	M182L	D279K	N253C-I284C [a]
H111E	D183F	Q289P [a]	Y260C-N265C
A113P	D183Y	M294R	N265-G268C [a]
W116E	R187F	A299P	H287C-H304C
W116D	D189P		

[a] the activity of the fermentation supernatant was too low, and thus the sample was not used for the following assay.

Table S2. Comparison of the DAG synthesis by using enzymes in this study and other reported enzymes via esterification.

enzyme	FA conversion (%)	MAG content (%)	1,3-DAG content (%)	1,2-DAG content (%)	Total DAG content (%)	TAG content (%)
Immobilized WT	71.40	31.17	26.57	13.66	40.23	\
Immobilized M5D	83.31	34.01	33.48	15.82	49.30	\
Novozyme 435 [38]	94.80	46.67	–	–	35.56	12.57
lipase G "Amano" 50 [39]	78.11	26.95	–	–	51.16	\
Immobilized PCL [8]	89.24	34.75	36.92	17.17	54.49	\

– represents that data are not reported. \ represents that TAGs cannot be detected.

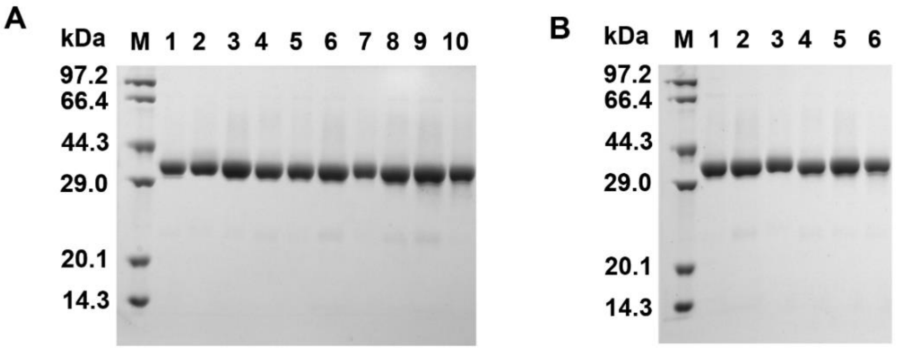

Figure S1. SDS-PAGE analysis of purified beneficial mutants (A) Lane M is the molecular mass marker. Lane 1 and 12 are Q34P, A37P, Y40W, M176I, M176V, G177A, T195L, T195I, T195F, R274P, D279K and M294R, respectively. (B) Lane M is the molecular mass marker. Lane 1 and 6 are G28C-P206C, M59C-T74C, G67C-V301C, S69C-S89C, I249C-A257C and H287C-H304C, respectively.

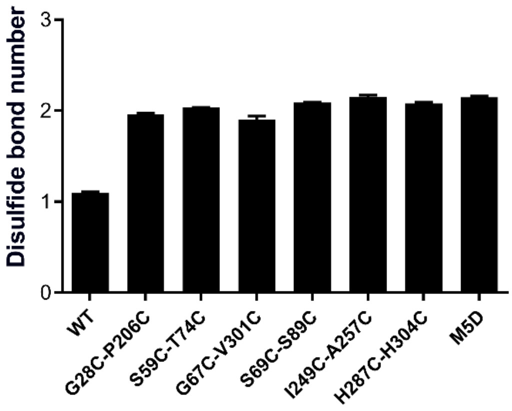

Figure S2. Disulfide bond number of the WT and beneficial mutant enzymes. Error bars represent standard deviations between replicates.

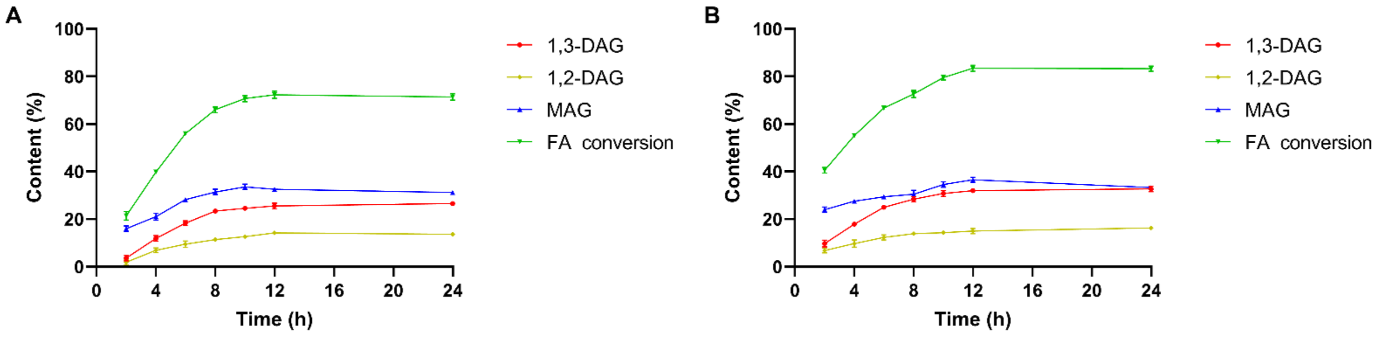

Figure S3. Compositions of the DAG synthesis reaction catalyzed by immobilized (A) WT and (B) M5D mutant. Error bars represent standard deviations between replicates.
